# Comparison of clinical features between patients with anti-synthetase syndrome and dermatomyositis: results from the MYONET registry

**DOI:** 10.1093/rheumatology/kead481

**Published:** 2023-09-12

**Authors:** Ryan Malcolm Hum, James B Lilleker, Janine A Lamb, Alexander G S Oldroyd, Guochun Wang, Lucy R Wedderburn, Louise P Diederichsen, Jens Schmidt, Maria Giovanna Danieli, Paula Oakley, Zoltan Griger, Thuy Nguyen Thi Phuong, Chanakya Kodishala, Monica Vazquez-Del Mercado, Helena Andersson, Boel De Paepe, Jan L De Bleecker, Britta Maurer, Liza McCann, Nicolo Pipitone, Neil McHugh, Robert Paul New, William E Ollier, Niels Steen Krogh, Jiri Vencovsky, Ingrid E Lundberg, Hector Chinoy, MYONET Registry, Sophie D’Hose, Xin Lu, Xiaolan Tian, Heřman Mann, Olga Kryštůfková, Lenka Pleštilová, Martin Klein, Tereza Barochová, Kateřina Kubínová, Chiara Gelardi, Alberto Paladini, Mario Andrea Piga, Luis J Jara, Miguel A Saavedra, Claudia V Cruz-Reyes, Olga Vera-Lastra, Lilia Andrade-Ortega, Gabriel Medrano-Ramírez, Minoru Satoh, Mario Salazar-Páramo, Efrain Chavarría-Ávila, Andrea Aguilar-Vazquez, Jesus-Aureliano Robles-de Anda, Marcelo H Petri, Øyvind Molberg, Maryam Dastmalchi, Antonella Notarnicola, Karina Gheorghe, Johan Rönnelid, Maria Liden, Balsam Hanna, Awat Jalal, Helena Hellström, Jehns Christian Martineus, Nguyen Thi Ngoc Lan, Leonid Padyukov, Paul New, Hazel Platt, Simon Rothwell, Yasmeen Ahmed, Raymond Armstrong, Robert Bernstein, Carol Black, Simon Bowman, Ian Bruce, Robin Butler, John Carty, Chandra Chattopadhyay, Easwaradhas Chelliah, Fiona Clarke, Peter Dawes, Christopher Denton, Joseph Devlin, Christopher Edwards, Paul Emery, John Fordham, Alexander Fraser, Hill Gaston, Patrick Gordon, Bridget Griffiths, Harsha Gunawardena, Frances Hall, Michael Hanna, Beverley Harrison, Elaine Hay, David Hilton-Jones, Lesley Horden, John Isaacs, David Isenberg, Adrian Jones, Sanjeet Kamath, Thomas Kennedy, George Kitas, Peter Klimiuk, Sally Knights, John Lambert, Peter Lanyon, Ramasharan Laxminarayan, Bryan Lecky, Raashid Luqmani, Pedro Machado, Jeffrey Marks, Michael Martin, Dennis McGonagle, Neil McHugh, Francis McKenna, John McLaren, Michael McMahon, Euan McRorie, Peter Merry, Sarah Miles, James Miller, Anne Nicholls, Jennifer Nixon, Voon Ong, Katherine Over, John Packham, Nicolo Pipitone, Michael Plant, Gillian Pountain, Thomas Pullar, Mark Roberts, Paul Sanders, David Scott, David Scott, Michael Shadforth, Thomas Sheeran, Arul Srinivasan, David Swinson, Lee-Suan Teh, Michael Webley, Brian Williams, Jonathan Winer

**Affiliations:** Centre for Musculoskeletal Research, Division of Musculoskeletal & Dermatological Sciences, The University of Manchester Faculty of Biology Medicine and Health, Manchester, UK; The University of Manchester, National Institute for Health Research Manchester Biomedical Research Centre, Manchester, UK; Centre for Musculoskeletal Research, Division of Musculoskeletal & Dermatological Sciences, The University of Manchester Faculty of Biology Medicine and Health, Manchester, UK; Northern Care Alliance NHS Foundation Trust, Manchester Centre for Clinical Neuroscience, Salford Royal Hospital, Salford, UK; Division of Population Health, Health Services Research and Primary Care, The University of Manchester Faculty of Biology Medicine and Health, Epidemiology and Public Health Group, Manchester, UK; Centre for Musculoskeletal Research, Division of Musculoskeletal & Dermatological Sciences, The University of Manchester Faculty of Biology Medicine and Health, Manchester, UK; The University of Manchester, National Institute for Health Research Manchester Biomedical Research Centre, Manchester, UK; Department of Rheumatology, China-Japan Friendship Hospital, Beijing, China; Great Ormond Street Hospital for Children NHS Foundation Trust, Infection, Immunity and Inflammation, London, UK; Center for Rheumatology and Spine Diseases, Copenhagen University Hospital, Copenhagen, Denmark; Department of Neurology, University Medical Center Göttingen, Göttingen, Germany; Department of Neurology and Pain Treatment, Neuromuscular Center, Center for Translational Medicine, Immanuel Klinik Rüdersdorf, University Hospital of the Brandenburg Medical School Theodor Fontane, Rüdersdorf bei Berlin, Germany; Faculty of Health Sciences Brandenburg, Brandenburg Medical School Theodor Fontane, Rüdersdorf bei Berlin, Germany; Clinica Medica, Dipartimento di Scienze Cliniche e Molecolari, Universita Politecnica delle Marche, Ancona, Italy; Myositis UK, Southampton, UK; Department of Immunology, University of Debrecen, Debrecen, Hajdú-Bihar, Hungary; Internal Medicine Department, Hanoi Medical University, Hanoi, Vietnam; Clinical Immunology and Rheumatology, St John's National Academy of Health Sciences, Bangalore, Karnataka, India; Department of Rheumatology, Mayo Clinic, Rochester, MN, USA; Division de Medicina Interna, Servicio de Reumatologia, Hospital Civil Dr. Juan I. Menchaca, Universidad de Guadalajara, Guadalajara, Jalisco, Mexico; Department of Rheumatology, Oslo University Hospital, Oslo, Norway; Department of Neurology, Universitair Ziekenhuis Gent, Ghent, Belgium; Department of Neurology, Universitair Ziekenhuis Gent, Ghent, Belgium; Department of Rheumatology and Immunology, Inselspital University Hospital Bern, Bern, Switzerland; Department of Rheumatology, Alder Hey Children's NHS Foundation Trust, Liverpool, UK; Department of Rheumatology, Arcispedale Santa Maria Nuova di Reggio Emilia, Reggio Emilia, Emilia-Romagna, Italy; Department of Rheumatology, Royal National Hospital for Rheumatic Diseases, Bath, UK; Department of Pharmacy and Pharmacology, University of Bath, Bath, UK; MRC/ARUK Institute of Ageing and Chronic Disease, University of Liverpool, Liverpool, UK; Faculty of Science and Engineering, Manchester Metropolitan University, Manchester, UK; Zitelab Aps, Copenhagen, Denmark; Institute of Rheumatology and Department of Rheumatology, Charles University, Praha, Czech Republic; Division of Rheumatology, Department of Medicine, Karolinska Institutet, Stockholm, Sweden; Department of Gastroenterology, Dermatology, and Rheumatology, Karolinska University Hospital, Stockholm, Sweden; Centre for Musculoskeletal Research, Division of Musculoskeletal & Dermatological Sciences, The University of Manchester Faculty of Biology Medicine and Health, Manchester, UK; Northern Care Alliance NHS Foundation Trust, Department of Rheumatology, Salford Royal Hospital, Salford, UK

**Keywords:** Anti-synthetase syndrome, Dermatomyositis, Cutaneous, Rashes, Skin, Malignancy, Epidemiology, MYONET, Extramuscular

## Abstract

**Objectives:**

To compare clinical characteristics, including the frequency of cutaneous, extramuscular manifestations and malignancy, between adults with anti-synthetase syndrome (ASyS) and DM.

**Methods:**

Using data regarding adults from the MYONET registry, a cohort of DM patients with anti-Mi2/-TIF1γ/-NXP2/-SAE/-MDA5 autoantibodies, and a cohort of ASyS patients with anti-tRNA synthetase autoantibodies (anti-Jo1/-PL7/-PL12/-OJ/-EJ/-Zo/-KS) were identified. Patients with DM *sine* dermatitis or with discordant dual autoantibody specificities were excluded. Sub-cohorts of patients with ASyS with or without skin involvement were defined based on presence of DM-type rashes (heliotrope rash, Gottron’s papules/sign, violaceous rash, shawl sign, V-sign, erythroderma, and/or periorbital rash).

**Results:**

In total 1054 patients were included (DM, *n* = 405; ASyS, *n* = 649). In the ASyS cohort, 31% (*n* = 203) had DM-type skin involvement (ASyS-DMskin). A higher frequency of extramuscular manifestations, including Mechanic’s hands, Raynaud’s phenomenon, arthritis, interstitial lung disease and cardiac involvement differentiated ASyS-DMskin from DM (all *P* < 0.001), whereas higher frequency of any of four DM-type rashes—heliotrope rash (*n* = 248, 61% *vs n* = 90, 44%), violaceous rash (*n* = 166, 41% *vs n* = 57, 9%), V-sign (*n* = 124, 31% *vs n* = 28, 4%), and shawl sign (*n* = 133, 33% *vs n* = 18, 3%)—differentiated DM from ASyS-DMskin (all *P* < 0.005). Cancer-associated myositis (CAM) was more frequent in DM (*n* = 67, 17%) compared with ASyS (*n* = 21, 3%) and ASyS-DMskin (*n* = 7, 3%) cohorts (both *P* < 0.001).

**Conclusion:**

DM-type rashes are frequent in patients with ASyS; however, distinct clinical manifestations differentiate these patients from classical DM. Skin involvement in ASyS does not necessitate increased malignancy surveillance. These findings will inform future ASyS classification criteria and patient management.

Rheumatology key messages

- Approximately one-third of patients with anti-synthetase syndrome have dermatomyositis-type cutaneous involvement.
- Certain clinical manifestations differentiate patients with anti-synthetase syndrome and dermatomyositis-type cutaneous involvement from dermatomyositis.
- Anti-synthetase syndrome with dermatomyositis-type cutaneous involvement is not associated with increased risk of malignancy.

## Introduction

Antisynthetase syndrome (ASyS) is a clinical subtype of idiopathic inflammatory myopathy (IIM) characterized by the presence of disease-specific autoantibodies against aminoacyl-transfer RNA synthetase (ARS) including anti-Jo1, -PL12, -PL7, -EJ, -OJ, -KS, -Zo and -Ha. Clinical features of ASyS include mechanic’s hands, Raynaud’s phenomenon, interstitial lung disease (ILD), myositis, arthritis and/or fever [1–3]. Dermatomyositis (DM) is another IIM subtype distinguished by characteristic cutaneous manifestations (including Gottron’s papules/sign, erythroderma, heliotrope, violaceous, periorbital, V-sign and shawl sign rashes) with or without myositis (amyopathic) and/or ILD [1]. DM-specific autoantibodies include anti-Mi2, -TIF1γ, -SAE, -MDA5 and -NXP2 [3]. Cutaneous DM-type manifestations can also be observed in ASyS patients, and therefore the current classification criteria for DM and ASyS overlap significantly, making classification of patients with anti-ARS and associated cutaneous manifestations especially challenging [4]. An international workshop from The European Neuromuscular Centre (ENMC) further highlighted this challenge, noting that ASyS is a unique and separate subgroup from DM even in the presence of DM-type cutaneous manifestations, and recommending that such patients be classified as having ‘ASyS with DM-like rash’ and not DM [5].

Up to 28% of patients with ASyS (defined with anti-ARS) have DM-type cutaneous manifestations [6]. However, it is not clear whether ASyS patients with DM-type cutaneous manifestations resemble patients with DM, and whether they should be regarded similarly in a clinical trial setting. Furthermore, it is not known if the presence of DM-type cutaneous manifestations confers an increased risk of DM-specific extramuscular manifestations, such as malignancy. Therefore, detailed phenotyping of a cohort of patients with ASyS with DM-type cutaneous manifestations might facilitate prediction of individual patient clinical course, clarify the need for malignancy screening, and inform future ASyS classification criteria.

We aimed to investigate the clinical manifestations in patients with ASyS and cutaneous manifestations using data from an international multicentre registry (MYONET registry, previously the EuroMyositis registry) [7].

## Methods

### The MYONET registry

The MYONET registry was created in 2003 [7]. The questions related to the registry were formulated following a Delphi process, and consensus discussion among Rheumatology and Neurology experts led to the creation of a uniform data collection proforma for use by all participating centres. Anonymized data from the registry were downloaded on 29 November 2021, which included 4806 cases from 112 centres, in 37 countries (Supplementary Table S1, available at *Rheumatology* online).

### ASyS and DM cohort definitions

As per registry inclusion criteria, all patients with DM met Bohan and Peter ‘definite’ or ‘probable’ diagnostic criteria [8], and all patients with ASyS met diagnostic criteria proposed by Connors *et al.* [9]. For this study, cohorts of patients with ASyS or DM were defined based on the presence of ARS or DM-specific autoantibodies [3]. Patients with any of the seven ARS autoantibodies (anti-Jo1, -PL12, -PL7, -EJ, -OJ, -Zo or -KS) detectable were defined as having ASyS, and patients with any of the five DM-specific autoantibodies (anti-Mi2, -TIF1γ, -SAE, -MDA5 or -NXP2) were defined as having DM. As Bohan and Peter diagnostic criteria for DM require cutaneous involvement, patients with DM *sine* dermatitis are not defined as DM in the registry. Five patients with both ARS and DM-specific autoantibodies were excluded. The presence of myositis-specific autoantibodies was reported by clinicians and results recorded within the registry. Methods for antibody testing varied depending on regional laboratory practices and were tabulated (Supplementary Table S2, available at *Rheumatology* online).

### Case characteristics

Patient demographics including sex, age at diagnosis, smoking status, autoantibodies, and clinical characteristics were collated. Clinical characteristics including the presence of myopathic muscle weakness, seven DM-type cutaneous manifestations (heliotrope rash, Gottron’s papules/sign, violaceous rash, erythroderma, periorbital rash, V-sign rash and shawl sign), 11 extramuscular manifestations (periungual erythema, calcinosis, ulceration, vasculitis, mechanic’s hands, Raynaud’s phenomenon, arthritis, dysphagia, alopecia, ILD and cardiac involvement), location and number of malignancies were recorded.

### Definition of ASyS with and without DM-type skin involvement sub-cohorts

Sub-cohorts of patients with ASyS with DM-type skin involvement (ASyS-DMskin) and those without DM-type skin involvement (ASyS-without-DMskin) were identified based on reported case characteristics. Patients with one or more of the DM-type cutaneous manifestation were considered to have DM-type skin involvement, and those with none considered without DM-type skin involvement. The sum of reported DM-type cutaneous manifestations out of a possible seven was calculated.

### Malignancy

Within the registry, malignancy is recorded including the date of diagnosis. In this analysis we considered malignancies diagnosed within 3 years of IIM onset to be ‘cancer-associated myositis’ (CAM). The location of CAM was compared between cohorts. Skin malignancies (including benign skin lesions such as basal cell carcinomas) were excluded except for melanoma. Malignancy was recorded variably by each centre, where in the UK the registry is linked to the National Health Service (NHS) Digital service that records malignancy, whereas other centres relied on entering malignancy data manually.

### Missing data

Comparing prevalence of the clinical manifestations in our cohort with previously reported data suggested that the data were missing not at random (MNAR), and that it was more likely that data were missing when the clinical characteristic was not present. Therefore, for statistical analysis imputation of missing values was considered inappropriate, and entries of clinical characteristics that were missing were considered not present. The number of missing entries for each clinical characteristic was tabulated (Supplementary Table S3, available at *Rheumatology* online).

### Statistical analysis

Between group comparisons were assessed using descriptive statistics as appropriate, with a threshold for significance set at *P* < 0.05. The Benjamini–Hochberg procedure was used to adjust for multiple comparisons to create adjusted *P*-values [10]. Statistical analyses were performed in R version 4.1.0 and RStudio version 1.4.1106 [11].

### Ethics

All patients gave informed written consent for their data to be analysed as part of this study. The MYONET (previously EuroMyositis) registry includes multiple recruiting centres in multiple countries, where ethical approvals are required and have been sought at each centre and informed consent is obtained from all included patients. All centres obtained specific ethical approval from their local ethics committees for this study.

## Results

### Case characteristics

**Figure 1.**
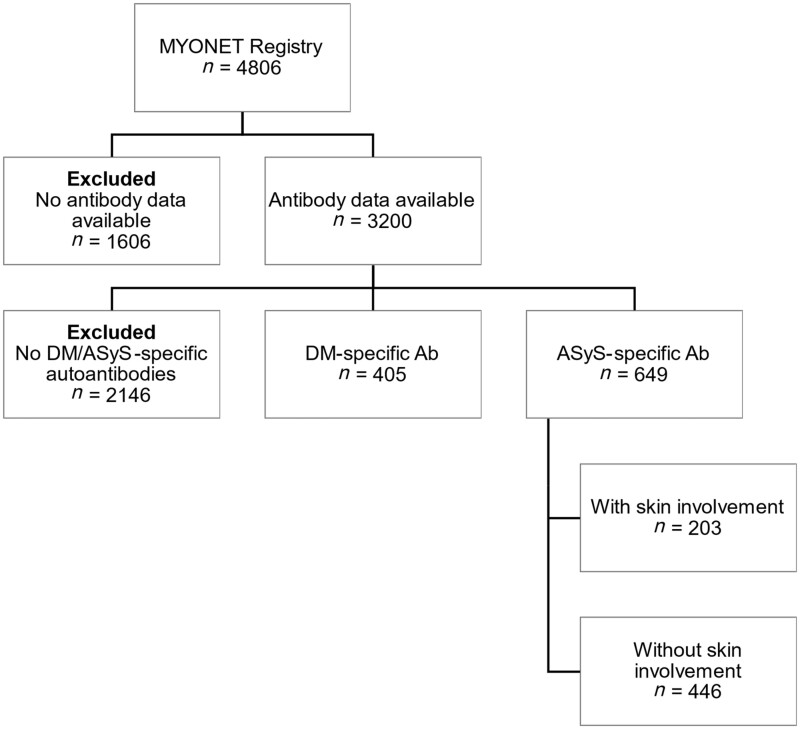
Flowchart illustrating the patients from the MYONET registry that were included and excluded from the study. DM-specific Ab refers to Mi2, TIF1γ, SAE, MDA5 and NXP2. ASyS-specific Ab refers to Jo1, PL12, PL7, EJ, OJ, Zo and KS. ASyS: anti-synthetase syndrome

Data regarding 4806 cases were initially analysed. Patients without results of autoantibody tests available were excluded (*n* = 1606) leaving 3200 cases (Supplementary Table S4, available at *Rheumatology* online). Of these, patients without ASyS or DM-specific autoantibodies (*n* = 2146) were excluded. A cohort of 405 patients with DM-specific autoantibodies was identified, while 649 patients with ARS autoantibodies were identified (Fig. 1).

### Demographics

Demographics including female sex, age at diagnosis and smoking status were compared between DM and ASyS groups. There was a significantly higher proportion of female sex in the ASyS-DMskin compared with the ASyS-without-DMskin cohorts (*n* = 147/203, 72% *vs n* = 278/446, 62%, *P* = 0.045). Age at diagnosis was significantly higher in the ASyS-without-DMskin cohort compared with the ASyS-DMskin cohort: 51 (interquartile range [IQR] 40–62) *vs* 47 (IQR 38–53) years, *P* = 0.005). Finally, there was a higher proportion of smokers in the ASyS cohort compared with the DM cohort (*n* = 197/649, 30% *vs n* = 96/405, 24%, *P* = 0.023) (Supplementary Table S5, available at *Rheumatology* online).

### Prevalence of disease-specific autoantibodies

The most common autoantibody in the DM cohort was anti-Mi2 (*n* = 162/405, 40%) followed by -TIF1γ (*n* = 143/405, 35%), -MDA5 (*n* = 66/405, 16%), -SAE (*n* = 39/405, 10%), and -NXP2 (*n* = 9/405, 2%) (Supplementary Table S6, available at *Rheumatology* online). In the ASyS cohort the majority possessed anti-Jo1 (*n* = 542/649, 84%) with a lower proportion possessing other ARS: anti-PL12 (*n* = 41/649, 6%), -PL7 (*n* = 35/649, 5%), -EJ (*n* = 16/649, 3%), -OJ (*n* = 10/649, 2%) and -Zo (*n* = 6/649, 1%) (Supplementary Table S6, available at *Rheumatology* online). There were no patients with anti-Ha antibodies recorded in the registry.

### Comparison of clinical characteristics between DM and ASyS cohorts

There were no significant differences in the presence of myopathic muscle weakness between DM and ASyS cohorts (Table 1). Patients in the DM cohort had a significantly higher frequency of each of the seven specified DM-type rashes compared with the ASyS cohort (Table 1). The extramuscular manifestations traditionally associated with ASyS (ILD, arthritis, Raynaud’s, mechanic’s hands) and cardiac involvement were predictably more common in this group compared with DM. Periungual erythema, ulceration, calcinosis, alopecia, vasculitis and dysphagia were more frequent in DM compared with ASyS, although there was overlap of these features across the two conditions (Table 1).

**Table 1.** Clinical manifestations of disease

					**Adjusted *P*-value** ^a^		
	DM (*n*=405)	ASyS (*n*=649)	ASyS-DMskin (*n*=203)	ASyS-without-DMskin (*n*=446)	DM vs ASyS	DM vs ASyS-DMskin	ASyS-DMskin *vs* ASyS-without-DMskin
Myopathic muscle weakness, *n* (%)	350 (86)	549 (85)	178 (88)	371 (83)	0.468	0.758	0.175
DM-type cutaneous manifestations, *n* (%)							
Heliotrope rash	248 (61)	90 (14)	90 (44)	0 (0)	<0.001	<0.001	
Gottron’s papules or sign	254 (63)	141 (22)	141 (70)	0 (0)	<0.001	0.152	
Violaceous rash	166 (41)	57 (9)	57 (28)	0 (0)	<0.001	0.004	
Erythroderma	37 (9)	15 (2)	15 (7)	0 (0)	<0.001	0.599	
Periorbital rash	97 (24)	38 (6)	38 (19)	0 (0)	<0.001	0.207	
V sign rash	124 (31)	28 (4)	28 (14)	0 (0)	<0.001	<0.001	
Shawl sign	133 (33)	18 (3)	18 (9)	0 (0)	<0.001	<0.001	
Extramuscular manifestations, *n* (%)							
Periungual erythema	148 (37)	110 (17)	56 (28)	54 (12)	<0.001	0.0503	<0.001
Calcinosis	22 (5)	13 (2)	9 (4)	4 (1)	0.0044	0.74	<0.001
Ulceration	28 (7)	8 (1)	4 (2)	4 (1)	<0.001	0.0272	0.0221
Vasculitis	11 (3)	2 (0.3)	0 (0)	2 (0.4)	0.0018	0.0552	0.533
Mechanic’s hands	45 (11)	200 (31)	84 (41)	116 (26)	<0.001	<0.001	<0.001
Raynaud’s phenomenon	55 (14)	252 (39)	90 (44)	162 (36)	<0.001	<0.001	0.109
Arthritis	64 (16)	312 (48)	101 (50)	211 (47)	<0.001	<0.001	0.679
Dysphagia	134 (33)	128 (20)	47 (23)	81 (18)	<0.001	<0.001	0.254
Alopecia	47 (12)	39 (6)	18 (9)	21 (5)	0.002	0.417	0.118
Interstitial lung disease	74 (18)	441 (68)	126 (62)	315 (71)	<0.001	<0.001	0.091
Cardiac involvement	9 (2)	46 (7)	19 (9)	27 (6)	<0.001	<0.001	0.233
CAM, *n* (%)	67 (17)	21 (3)	7 (3)	14 (3)	<0.001	<0.001	1

a Chi-square test. ASyS: antisynthetase syndrome; ASyS-DMskin: antisynthetase syndrome with skin involvement; ASyS-without-DMskin: antisynthetase syndrome without skin involvement; CAM: cancer-associated myositis.

### ASyS with DM-type skin involvement sub-cohort and comparison of clinical characteristics with DM cohort

The DM cohort was compared with ASyS patients possessing DM-type rashes. Of the 649 patients in the ASyS cohort, 31% (*n* = 203/649) had at least one of the seven DM-type rashes indicating skin involvement. Heliotrope rash, violaceous rash, V-sign and shawl sign were significantly more frequent in the DM cohort compared with the ASyS-DMskin sub-cohort, whereas there was no difference in frequency between DM and ASyS-DMskin for the remaining three DM-type rashes (Gottron’s papules/sign, periorbital rash, erythroderma). As was observed in the overall ASyS cohort, ILD, arthritis, Raynaud’s, mechanic’s hands and cardiac involvement were significantly more frequent in the ASyS-DMskin sub-cohort, compared with the DM cohort. However, there were no significant differences in the frequency of myopathic muscle weakness, periungual erythema, calcinosis, vasculitis and alopecia in the ASyS-DMskin and DM cohorts. (Table 1).

For the DM cohort, the median number of DM-type rashes reported was 2 out of 7 (IQR 1–4), which was significantly higher than the overall ASyS cohort (median 0, IQR 0–1, *P* < 0.001) and comparable to the ASyS-DMskin sub-cohort (median 2, IQR 1–2, *P* < 0.001) (Supplementary Table S7, available at *Rheumatology* online).

A comparison of extramuscular manifestations between the ASyS-DMskin and ASyS-without-DMskin sub-cohorts showed that the frequency of periungual erythema, calcinosis, mechanic’s hands and ulceration was significantly higher in the ASyS-DMskin sub-cohort (Table 1).

### Comparison of clinical characteristics in ASyS and in DM by antibody

In patients with ASyS, DM-type cutaneous manifestations were seen in 25% (*n* = 136/542) of those with anti-Jo1, 27% (*n* = 11/41) with -PL12, 23% (*n* = 8/35) with -PL7, 19% (*n* = 3/16) with -EJ, 40% (*n* = 4/10) with -OJ and 0% (*n* = 0/6) with -Zo antibodies (Supplementary Table S8, available at *Rheumatology* online). The frequency of myopathic muscle weakness, arthritis and dysphagia within the ASyS cohort was not equally distributed across the different anti-ARS antibody subtypes where the lowest frequency of myopathic muscle weakness was seen in those with anti-PL12 antibodies (46%, *n* = 19/41), and the highest frequency of arthritis and dysphagia was seen in those anti-Zo antibodies (67%, *n* = 4/6 and 50%, *n* = 3/6, respectively) (Supplementary Table S8, available at *Rheumatology* online). The frequency of periungual erythema, ulceration, mechanic’s hands, arthritis, dysphagia, alopecia and ILD, as well as the frequency of certain DM-type cutaneous manifestations (heliotrope rash, Gottron’s papules/sign, violaceous rash, periorbital rash and V-sign rash) within the DM cohort were not equally distributed across DM antibody subtypes (Supplementary Table S9, available at *Rheumatology* online). In those with anti-MDA5 antibodies, there was high frequency of extramuscular manifestations including calcinosis (13%, *n* = 8/63), mechanic’s hands (27%, *n* = 17/63), arthritis (38%, *n* = 24/63) and ILD (57%, *n* = 36/63). Cutaneous manifestations were generally more frequent in those with anti-TIF1γ antibodies and in those with anti-SAE antibodies and less frequent in those with anti-MDA5 and anti-Mi2 antibodies.

### Comparison of CAM in disease cohorts and by antibody

The number of patients with at least one CAM was significantly higher in the DM cohort compared with the ASyS cohort (*n* = 67/405, 17% *vs n* = 21/649, 3%, *P*_adjusted_ < 0.001), and in the DM cohort compared with the ASyS-DMskin cohort (*n* = 67/405, 17% *vs n* = 7/203, 3%, *P*_adjusted_ < 0.001) (Table 1). There was no significant difference between the frequency of CAM in ASyS-DMskin compared with ASyS-without-DMskin cohorts (*n* = 7/203, 3% *vs n* = 14/446, 3%, *P*_adjusted_ = 1) (Table 1).

Bowel (12/405, 3% *vs* 2/649, 0.3%, *P*_adjusted_ = 0.013), breast (16/405, 4% *vs* 7/649, 1%, *P*_adjusted_ = 0.02), lung (10/405, 3% *vs* 3/649, 0.5%, *P*_adjusted_ = 0.03) and ovarian cancers (15/405, 4% *vs* 0/649, 0%, *P*_adjusted_ = 0.007) were more frequently reported in DM compared with ASyS (Supplementary Table S10, available at *Rheumatology* online). There were no significant differences in location of CAM between DM and ASyS-DMskin, or between ASyS-DMskin and ASyS-without-DMskin cohorts (Supplementary Table S10, available at *Rheumatology* online). The frequency of CAM was not equally distributed between antibody subtypes, χ^*2*^ (degrees of freedom = 9, *n* = 737, *P*_adjusted_ < 0.001), and notably the highest frequency of CAM was observed in anti-TIF1γ patients (33%, *n* = 46/138) (Supplementary Table S11, available at *Rheumatology* online).

## Discussion

We identified several important findings including: (i) one-third of ASyS patients have DM-type cutaneous manifestations; (ii) DM-specific skin rashes in ASyS patients were associated with a distinct phenotype including higher frequency of mechanic’s hands, Raynaud’s phenomenon, arthritis, ILD and cardiac involvement and lower frequency of ulceration and dysphagia; and (iii) DM-specific skin rash in ASyS patients was not associated with increased risk of cancer.

First, our study demonstrates that a third of patients with ASyS have DM-type cutaneous manifestations. Our results are consistent with the previous largest published study (*n* = 233), which found DM-type cutaneous manifestations with a prevalence of 28% in patients with ASyS [6]. This confirms that DM-type cutaneous manifestations are observed in a substantial proportion of patients with ASyS. Interestingly, our cohort also includes patients with EJ, OJ and Zo antibodies, whereas the previous study included patients with Jo1, PL12 and PL7 [6]. Our study therefore supports previous notions that a large proportion of ASyS patients have DM-specific skin manifestations, regardless of autoantibody status. Clinicians should therefore be vigilant for DM-specific manifestations in ASyS patients and actively treat them due to their detrimental impact on quality of life [12].

Second, our study demonstrates that DM-specific rashes in ASyS patients are associated with a distinct phenotype that differentiates them from DM and from ASyS patients without DM-specific rashes. However, we also noted that increased frequency of cardiac involvement differentiated ASyS from DM, and that increased frequency of mechanic’s hands, calcinosis, ulceration and periungual erythema differentiates ASyS-DMskin from ASyS-without-DMskin, suggesting that the pathogenesis underlying ASyS-specific cutaneous manifestations may have additional vascular and endothelial aetiologies over and above that which is seen in DM-specific cutaneous manifestations. We identified clinical features including increased frequency of mechanic’s hands, Raynaud’s phenomenon, arthritis, cardiac involvement and ILD that differentiate ASyS-DMskin from DM. Therefore, clinicians should consider a diagnosis of ASyS if these clinical signs are noted in the presence of DM-type rashes. Conversely, certain DM-type rashes (heliotrope rash, V-sign, violaceous rash and shawl sign) differentiate DM from ASyS-DMskin, and were infrequently observed in ASyS. Therefore, clinicians may not need to prioritize ASyS highly in the presence of these DM-type rashes and should instead prioritize a diagnosis of DM, and ensure malignancy screening and that other disease-specific management considerations are appropriately targeted.

Third, our study assesses whether ASyS-DMskin is associated with an increased risk of CAM and found that CAM was more frequent in DM compared with ASyS, as previously reported, but that CAM was not more frequent in ASyS-DMskin compared with ASyS-without-DMskin. The surveillance of malignancy is vital in the clinical management of DM given that it is the main cause of death in patients with IIM [14]. Interestingly, ILD and presence of anti-ARS have been associated with a lower risk of CAM, suggesting that patients with ASyS may have reduced risk of CAM compared with other IIM subtypes such as DM [1, 15]. Our findings suggest that although the cutaneous manifestations in ASyS-DMskin may be driven by similar biological processes as in DM, in ASyS-DMskin this may not confer increased risk of CAM. Therefore, in clinical practice, skin involvement in ASyS need not prompt increased surveillance or investigation for CAM.

The main strength of our study is the use of international registry data which includes the largest reported cohort of patients with DM and ASyS representing patients from centres around the world with different ethnicities. This is important given that DM and ASyS are rare diseases and would be otherwise difficult to study. However, use of registry data has limitations. First, missing data is an issue which may affect the accuracy of our findings. Second, although international collaboration is a strength when studying rare diseases, variations in clinical practice may lead to variability in reporting across centres. Third, although all patients in the MYONET registry have met current IIM classification criteria, we have further defined our DM and ASyS cohorts based on the presence of autoantibodies. However, not all patients with IIM have identifiable autoantibodies, for example, one study found 28% of DM cases were seronegative, and certain rare ASyS antibodies cannot be tested for in routine clinical practice and are therefore not represented in our study [16]. Fourth, the registry relies on clinicians with an expertise in IIM to apply IIM classification criteria prior to inclusion, and case notes were not reviewed or verified, potentially introducing a degree of misclassification. Fifth, the data analysed in this study are cross-sectional meaning clinical features that develop after entry to the registry are not captured. Finally, our analysis makes no comparison with healthy or connective tissue disease populations. Therefore, we cannot draw conclusions about whether frequency of malignancy in ASyS is higher than in the general population.

In conclusion, this is the largest study to date comparing clinical manifestations in ASyS to DM, and the first study to specifically investigate a cohort with ASyS and skin manifestations akin to DM. A third of patients with ASyS have DM-type cutaneous involvement compatible with a diagnosis of DM, but although this cohort resembles DM in terms of skin rashes, there are specific clinical manifestations which differentiate the two, and risk of CAM is lower than DM and similar to ASyS patients without DM-type skin involvement. Work to elucidate the biological processes underlying clinical manifestations in these cohorts would improve our ability to classify patients and develop targeted treatments for specific disease manifestations. These findings can inform future ASyS classification criteria and improve our ability to classify patients and develop targeted treatments for specific disease manifestations.

## Supplementary Material

kead481_Supplementary_Data

## Data Availability

Data will be shared upon reasonable requests to the corresponding author.
