# Correction to: Comparison of clinical features between patients with anti-synthetase syndrome and dermatomyositis: results from the MYONET registry

**DOI:** 10.1093/rheumatology/keae186

**Published:** 2024-03-26

This is a correction to: Ryan Malcolm Hum, James B Lilleker, Janine A Lamb, Alexander G S Oldroyd, Guochun Wang, Lucy R Wedderburn, Louise P Diederichsen, Jens Schmidt, Maria Giovanna Danieli, Paula Oakley, Zoltan Griger, Thuy Nguyen Thi Phuong, Chanakya Kodishala, Monica Vazquez-Del Mercado, Helena Andersson, Boel De Paepe, Jan L De Bleecker, Britta Maurer, Liza McCann, Nicolo Pipitone, Neil McHugh, Robert Paul New, William E Ollier, Niels Steen Krogh, Jiri Vencovsky, Ingrid E Lundberg, Hector Chinoy, MYONET Registry, Comparison of clinical features between patients with anti-synthetase syndrome and dermatomyositis: results from the MYONET registry, *Rheumatology*, 2023; kead481, https://doi.org/10.1093/rheumatology/kead481

In the originally published version of this manuscript two affiliations for the author Jens Schmidt were inadvertently omitted:

1. Department of Neurology and Pain Treatment, Neuromuscular Center, Center for Translational Medicine, Immanuel Klinik Rüdersdorf, University Hospital of the Brandenburg Medical School Theodor Fontane, Rüdersdorf bei Berlin, Germany
2. Faculty of Health Sciences Brandenburg, Brandenburg Medical School Theodor Fontane, Rüdersdorf bei Berlin, Germany

This error has been corrected in the original paper.

