# Supplementary material for: Comparison of clinical features between patients with anti-synthetase syndrome and dermatomyositis: results from the MYONET registry
Source: Rheumatology (Oxford). 2023 Sep 12;63(8):2093–100. doi: 10.1093/rheumatology/kead481 (PMC11292049; doi:10.1093/rheumatology/kead481)
Supplement: kead481_Supplementary_Data [file kead481_supplementary_data.docx]

**Supplementary Materials**

| **Supplementary Table S1** | Centres with corresponding countries represented in the MYONET registry |
| --- | --- |
| **Supplementary Table S2** | Antibody Testing Techniques |
| **Supplementary Table S3** | Missing data by cohort |
| **Supplementary Table S4** | Country of patients with autoantibody data available for analysis |
| **Supplementary Table S5** | Demographics by disease cohort |
| **Supplementary Table S6** | Autoantibody subtypes |
| **Supplementary Table S7** | Number of DM-type cutaneous manifestations by disease cohort |
| **Supplementary Table S8** | Clinical manifestations of ASyS cohort by antibody |
| **Supplementary Table S9** | Clinical manifestations of DM cohort by antibody |
| **Supplementary Table S10** | Malignancies by disease cohort |
| **Supplementary Table S11** | Malignancies by antibody cohort |

**Supplementary Table S1** Centres with corresponding countries represented in the MYONET registry

| **#** | **Hospital Name** | **Country** |
| --- | --- | --- |
| 1 | CEMIC (University Hospital) Buenos Aires, Argentina | Argentina |
| 2 | Landeskrankenhaus Innsbruck, Innsbruck Medical University | Australia |
| 3 | Hanusch Hospital | Australia |
| 4 | University of Adelaide | Australia |
| 5 | Austin Health | Australia |
| 6 | Fiona Stanley Hospital, Neurology | Australia |
| 7 | Department of Rheumatology, Royal Prince Alfred Hospital, Sydney | Australia |
| 8 | University of Gent | Belgium |
| 9 | UZ Leuven | Belgium |
| 10 | University Hospital Ghent, Department of Rheumatology / Clinical Biology | Belgium |
| 11 | Faculdade de Medicina da Universidade de Sao Paulo | Brazil |
| 12 | St Michaels Hospital | Canada |
| 13 | Jewish General Hospital, Montreal | Canada |
| 14 | University Hospital Zurich | Switzerland |
| 15 | University of Chile, Clinical Hospital, Rheumatology Section | Chile |
| 16 | China-Japan Friendship Hospital, Department of Rheumatology | China |
| 17 | Neurology department, University of Cyprus | Cyprus |
| 18 | Prague Hospital | Czechia |
| 19 | Klinika pediatrie a dedicnych poruch metabolismu (Prague) | Czechia |
| 20 | Humboldt University | Germany |
| 21 | University of Goettingen | Germany |
| 22 | University of Dresden | Germany |
| 23 | University of Bonn | Germany |
| 24 | Uniklinik Munster | Germany |
| 25 | Department of Rheumatology and Immunology, Erlangen | Germany |
| 26 | Center for autoimmune diseases | Spain |
| 27 | Hospital Universitari Vall d'Hebron | Spain |
| 28 | Hospital barcelona | Spain |
| 29 | Cruces University Hospital | Spain |
| 30 | Hospital Sant Joan de Deu, Barcelona, Pediatric Rheumatology | Spain |
| 31 | Department of Rheumatology, Ramon y Cajal University Hospital, Madrid | Spain |
| 32 | University of Eastern Finland | Finland |
| 33 | Hopitaux Universitaires Pitie Salpetriere, Centre de reference neuromusculaire | France |
| 34 | Hopital Universitaire Necker-Enfants Malades, Paris | France |
| 35 | Salford Royal NHS Foundation Trust | United Kingdom |
| 36 | Kings College, London | United Kingdom |
| 37 | Royal United Hospitals Bath NHS Foundation Trust | United Kingdom |
| 38 | Aintree University Hospital NHS FT | United Kingdom |
| 39 | Basildon & Thurrock University Hospitals FT | United Kingdom |
| 40 | Burton Hospitals NHS FT | United Kingdom |
| 41 | Cambridge University Hospitals NHS FT (Addenbrookes Hospital) | United Kingdom |
| 42 | Doncaster and Bassetlaw Hospitals NHS FT (Doncaster Royal Infirmary) | United Kingdom |
| 43 | James Paget University Hospital NHS FT (James Paget University Hospital) | United Kingdom |
| 44 | London North West Healthcare NHS Trust (Northwick Park Hospital) | United Kingdom |
| 45 | North Bristol NHS Trust (Southmead Hospital) | United Kingdom |
| 46 | Northampton General Hospital NHS Trust (Northampton General Hospital) | United Kingdom |
| 47 | Nottingham University Hospitals NHS Trust | United Kingdom |
| 48 | Royal Free London NHS FT (Royal Free Hospital) | United Kingdom |
| 49 | Sandwell & West Birmingham Hospitals NHS Trust (City Hospital) | United Kingdom |
| 50 | Sheffield Teaching Hospital NHS FT (Royal Hallamshire Hospital) | United Kingdom |
| 51 | St George's University Hospitals NHS FT | United Kingdom |
| 52 | The Dudley Group NHS FT (Russells Hall Hospital) | United Kingdom |
| 53 | The Newcastle Upon Tyne Hospitals NHS Trust (Royal Victoria Infirmary) | United Kingdom |
| 54 | University College London NHS FT (Centre for Rheumatology & UCL division of Medicine) | United Kingdom |
| 55 | University College London NHS FT (The National Hospital for Neurology and Neurosurgery) | United Kingdom |
| 56 | University Hospitals of Birmingham NHS FT (Solihull Hospital) | United Kingdom |
| 57 | University Hospitals of Derby and Burton NHS Foundation Trust (Royal Derby Hospital) | United Kingdom |
| 58 | Queen Elizabeth Hospital - Birmingham | United Kingdom |
| 59 | Leighton Hospital - Crewe | United Kingdom |
| 60 | Lancaster Infirmary - Morecambe Bay | United Kingdom |
| 61 | St Helens and Knowsley NHS Foundation Trust | United Kingdom |
| 62 | Stockport NHS Foundation trust | United Kingdom |
| 63 | The Alexandra Hospital | United Kingdom |
| 64 | Bradford Teaching Hospital | United Kingdom |
| 65 | Frimley Park Hospital | United Kingdom |
| 66 | University Hospital Coventry & Warwickshire | United Kingdom |
| 67 | Gateshead Health NHS Foundation Trust (Queen Elizabeth Hospital) | United Kingdom |
| 68 | Royal Freeman Hospital (Newcastle) | United Kingdom |
| 69 | Rheumatology center at Laikon University Hospital, Athens | Greece |
| 70 | Kwong Wah Hospital, Rheumatology | Hong Kong |
| 71 | University of Debrecen | Hungary |
| 72 | Rheumatology Department, Budapest | Hungary |
| 73 | SAOLTA University Healthcare Group | Ireland |
| 74 | Department of Clinical Immunology, S.G.P.G.I.M., Lucknow, India | India |
| 75 | Tel Aviv Medical Center | Israel |
| 76 | Rheumatology Department, Rambam Health Care Campus (Haifa) | Iceland |
| 77 | Arcispedale Santa Maria Nuova | Italy |
| 78 | Clinica Medica, Dipartimento di Scinze Mediche e Chirurgiche, Polo Didattico Scientifico | Italy |
| 79 | University of Padova | Italy |
| 80 | University of Milan | Italy |
| 81 | Rheumatology Department of Regina Margherita Children Hospital | Italy |
| 82 | San Raffaele Scientific Institute, Milan, Italy | Italy |
| 83 | Anna Meyer Childrens Hospital, Italy | Italy |
| 84 | University of Siena | Italy |
| 85 | Tokyo Medical and Dental University | Japan |
| 86 | Seoul National University Hospital | Korea |
| 87 | Vilnius University Hospital Santaros Clinics Affiliate Children Hospital | Lithuania |
| 88 | Hospital Civil de Guadalajara | Mexico |
| 89 | Rheumatology and Immunology department, Instituto Nacional de Ciencias Medicas y Nutricion Salvador Zubiran | Mexico |
| 90 | University of Amsterdam | Netherlands |
| 91 | Radboud University Medical Centre | Netherlands |
| 92 | Utrecht University Hospital | Netherlands |
| 93 | Leiden University Medical Centre | Netherlands |
| 94 | Oslo University Hospital Reuma Adult unit | Norway |
| 95 | Oslo University Hospital Reuma Pediatric Unit | Norway |
| 96 | Institute of Rheumatology (Warsaw) | Poland |
| 97 | Narodowy Instytut Geriatrii, Reumatologii i Rehabilitacji im. prof. dr hab. med. Eleonory Reicher | Poland |
| 98 | USK-WAM Clinical Hospital in Lodz | Poland |
| 99 | Cabral/Centro Hospitalar Lisboa Central | Portugal |
| 100 | Porto Hospital | Portugal |
| 101 | Karolinska Hospital | Sweden |
| 102 | Sahlgrenska University Hospital | Sweden |
| 103 | Uppsala University hospital | Sweden |
| 104 | Vastmanlands hospital | Sweden |
| 105 | Rheumatology at Orebro University Hospital | Sweden |
| 106 | Sidi Bouzid Regional Hospital, | Tunisia |
| 107 | Uludag University Medical Faculty | Turkey |
| 108 | Hacettepe University Faculty of Medicine, Ankara | Turkey |
| 109 | University of Kentucky | United States of America |
| 110 | New York Methodist Hospital | United States of America |
| 111 | Division of Rheumatology, Loma Linda Universisity | United States of America |
| 112 | Hanoi Medical University | Vietnam |

**Supplementary Table S2** Antibody Testing Techniques

| **Technique** | **n /1054 (%)** |
| --- | --- |
| Immunoprecipitation | 587 (56) |
| Lineblot | 238 (23) |
| ELISA | 174 (17) |
| Other/Unknown | 55 (5) |

**Supplementary Table S3** Missing data by cohort

|  | **DM cohort**  **n=405 (%)** | **ASyS cohort**  **n=649 (%)** |
| --- | --- | --- |
| **Myopathic Muscle Weakness** | 24 (6) | 32 (5) |
| **DM-type cutaneous manifestations** |  |  |
| Heliotrope Rash | 37 (9) | 143 (22) |
| Gottron’s Papules or Sign | 41 (10) | 138 (21) |
| Violaceous Rash | 63 (16) | 168 (26) |
| Erythroderma | 176 (43) | 337 (52) |
| Periorbital Rash | 171 (42) | 334 (46) |
| V Sign Rash | 108 (27) | 299 (46) |
| Shawl Sign | 107 (26) | 311 (48) |
| **Extramuscular manifestations** |  |  |
| Periungual Erythema | 149 (37) | 286 (44) |
| Calcinosis | 146 (36) | 302 (47) |
| Ulceration | 162 (40) | 336 (52) |
| Vasculitis | 172 (42) | 381 (59) |
| Mechanic’s Hands | 91 (22) | 143 (22) |
| Raynaud’s Phenomenon | 129 (32) | 132 (20) |
| Arthritis | 38 (9) | 53 (8) |
| Dysphagia | 79 (20) | 159 (24) |
| Alopecia | 141 (35) | 304 (47) |
| Interstitial Lung Disease | 31 (8) | 22 (3) |
| Cardiac Involvement | 115 (28) | 205 (32) |

**Supplementary Table S4** Country of patients with autoantibody data included in the analysis

| **Country** | **Cases with autoantibody data available (/1054) n(%)** |
| --- | --- |
| Argentina | 15 (1) |
| Belgium | 8 (0.8) |
| Brazil | 14 (1) |
| Czechia | 106 (10) |
| Germany | 9 (0.9) |
| Hungary | 16 (2) |
| Italy | 46 (4) |
| Mexico | 80 (8) |
| Netherlands | 2 (0.2) |
| Poland | 2 (0.2) |
| Sweden | 306 (29) |
| Turkey | 2 (0.2) |
| United Kingdom | 448 (43) |

**Supplementary Table S5** Demographics by disease cohort

|  | **DM (n=405)** | **ASyS (n=649)** | **ASyS-DMskin**  **(n=203)** | **ASyS-without-DMskin**  **(n=446)** | **DM vs ASyS p-value** | **DM vs ASyS- DMskin p-value** | **ASyS-DMskin vs ASyS-without-DM skin p-value** |
| --- | --- | --- | --- | --- | --- | --- | --- |
| Female sex | 257 (64%) | 425 (66%) | 147 (72%) | 278 (62%) | 1 | 0.186 | 0.045 |
| Age at diagnosis, median (IQR) | 50 (36 to 64) | 49 (39 to 60) | 47 (38 to 53) | 51 (40 to 62) | 0.795 | 0.093 | 0.005 |
| Smoker | 96 (24%) | 197 (30%) | 63 (31%) | 134 (30%) | 0.023 | 0.066 | 0.871 |

**Supplementary Table S6** Autoantibody subtypes

| **Autoantibody** | **DM (n=405)** | **ASyS (n=649)** | **ASyS-DMskin**  **(n=203)** | **ASyS-without-DMskin**  **(n=446)** |
| --- | --- | --- | --- | --- |
| MI2 | 162 (40%) | 0 | | |
| TIF1γ | 143 (35%) |  |  |  |
| SAE | 39 (10%) |  |  |  |
| MDA5 | 66 (16%) |  |  |  |
| NXP2 | 9 (2%) |  |  |  |
| Jo1 | 0 | 541 (84%) | 136 (83.4%) | 369 (83%) |
| PL12 |  | 41 (6%) | 11 (6.7%) | 28 (6%) |
| PL7 |  | 35 (5%) | 8 (4.9%) | 25 (6%) |
| EJ |  | 16 (3%) | 3 (1.8%) | 13 (3%) |
| OJ |  | 10 (2%) | 4 (2.5%) | 6 (1%) |
| Zo |  | 6 (1%) | 0 | 6 (1%) |
| KS |  | 0 | 0 | 0 |

**Supplementary Table S7** Number of DM-type cutaneous manifestations by disease cohort

|  | **# of rashes,** median (IQR) |
| --- | --- |
| **DM (n=405)** | 2 (1-4) |
| **ASyS (n=649)** | 0 (0-1) |
| **ASyS-DMskin (n=203)** | 2 (1 to 2) |
| **DM vs ASyS p-value** | <0.001 |
| **DM vs ASyS-DMskin p-value** | <0.001 |

**Supplementary Table S8** Clinical manifestations of ASyS cohort by antibody

|  | **EJ (n=16)** | **Jo1 (n=541)** | **OJ (n=10)** | **PL12 (n=41)** | **PL7 (n=35)** | **Zo (n=6)** | **Adjusted**  **p-value^1^** |
| --- | --- | --- | --- | --- | --- | --- | --- |
| **Myopathic Muscle Weakness n (%)** | 12 (75) | 474 (88) | 10 (100) | 19 (46) | 28 (80) | 6 (100) | <0.001 |
| **DM-type cutaneous manifestations n (%)** |  |  |  |  |  |  |  |
| Heliotrope Rash | 1 (6) | 73 (13) | 3 (30) | 6 (14) | 7 (20) | 0 (0) | 0.851 |
| Gottron’s Papules or Sign | 2 (12) | 122 (22) | 2 (20) | 9 (22) | 6 (17) | 0 (0) | 0.860 |
| Violaceous Rash | 2 (12) | 46 (8) | 3 (30) | 4 (9) | 2 (5) | 0 (0) | 0.703 |
| Erythroderma | 0 (0) | 13 (2) | 0 (0) | 1 (2) | 1 (2) | 0 (0) | 1.09 |
| Periorbital Rash | 0 (0) | 36 (6) | 0 (0) | 2 (4) | 0 (0) | 0 (0) | 0.748 |
| V Sign Rash | 0 (0) | 25 (4) | 0 (0) | 0 (0) | 3 (8) | 0 (0) | 0.891 |
| Shawl Sign | 0 (0) | 17 (3) | 0 (0) | 0 (0) | 1 (2) | 0 (0) | 0.948 |
| **Extramuscular manifestations n (%)** |  |  |  |  |  |  |  |
| Periungual Erythema | 1 (6) | 92 (17) | 0 (0) | 11 (26) | 4 (11) | 2 (33) | 0.574 |
| Calcinosis | 0 (0) | 12 (2) | 0 (0) | 1 (2) | 0 (0) | 0 (0) | 0.960 |
| Ulceration | 0 (0) | 6 (1) | 0 (0) | 2 (4) | 0 (0) | 0 (0) | 1.01 |
| Vasculitis | 0 (0) | 2 (0) | 0 (0) | 0 (0) | 0 (0) | 0 (0) | 0.995 |
| Mechanic’s Hands | 4 (25) | 168 (31) | 0 (0) | 20 (48) | 6 (17) | 2 (33) | 0.010 |
| Raynaud’s Phenomenon | 3 (18) | 212 (39) | 1 (10) | 16 (39) | 16 (45) | 4 (66) | 0.537 |
| Arthritis | 6 (37) | 280 (51) | 2 (20) | 15 (36) | 5 (14) | 4 (66) | <0.001 |
| Dysphagia | 2 (12) | 112 (20) | 1 (10) | 0 (0) | 10 (28) | 3 (50) | 0.048 |
| Alopecia | 3 (18) | 33 (6) | 0 (0) | 1 (2) | 1 (2) | 1 (16) | 0.497 |
| Interstitial Lung Disease | 11 (68) | 373 (68) | 4 (40) | 28 (68) | 21 (60) | 4 (66) | 0.686 |
| Cardiac Involvement | 0 (0) | 43 (7) | 0 (0) | 2 (4) | 1 (2) | 0 (0) | 0.740 |
| ^1^Chi-squared test | | | | | | | |

**Supplementary Table S9** Clinical manifestations of DM cohort by antibody

|  | **MDA5 (n=63)** | **Mi2 (n=160)** | **NXP2 (n=9)** | **SAE (n=35)** | **TIF1γ (n=138)** | **Adjusted p-value^1^** |
| --- | --- | --- | --- | --- | --- | --- |
| **Myopathic Muscle Weakness n (%)** | 48 (76) | 142 (89) | 8 (89) | 32 (91) | 120 (87) | 0.126 |
| **DM-type cutaneous manifestations n (%)** |  |  |  |  |  |  |
| Heliotrope Rash | 31 (49) | 86 (53) | 5 (55) | 25 (71) | 101 (73) | 0.004 |
| Gottron’s Papules or Sign | 42 (66) | 83 (51) | 3 (33) | 27 (77) | 99 (71) | <0.001 |
| Violaceous Rash | 30 (47) | 49 (30) | 3 (33) | 16 (45) | 68 (49) | 0.022 |
| Erythroderma | 8 (12) | 10 (6) | 0 (0) | 3 (8) | 16 (11) | 0.409 |
| Periorbital Rash | 13 (20) | 24 (15) | 3 (33) | 10 (28) | 47 (34) | 0.007 |
| V Sign Rash | 14 (22) | 39 (24) | 3 (33) | 12 (34) | 56 (40) | 0.031 |
| Shawl Sign | 14 (22) | 51 (31) | 1 (11) | 13 (37) | 54 (39) | 0.117 |
| **Extramuscular manifestations n (%)** |  |  |  |  |  |  |
| Periungual Erythema | 33 (52) | 37 (23) | 2 (22) | 15 (42) | 61 (44) | <0.001 |
| Calcinosis | 8 (12) | 6 (3) | 1 (11) | 3 (8) | 4 (2) | 0.052 |
| Ulceration | 11 (17) | 5 (3) | 0 (0) | 3 (8) | 9 (6) | 0.009 |
| Vasculitis | 2 (3) | 4 (2) | 0 (0) | 0 (0) | 5 (3) | 0.828 |
| Mechanic’s Hands | 17 (27) | 11 (6) | 1 (11) | 1 (2) | 15 (10) | <0.001 |
| Raynaud’s Phenomenon | 9 (14) | 29 (18) | 0 (0) | 1 (2) | 16 (11) | 0.117 |
| Arthritis | 24 (38) | 20 (12) | 2 (22) | 5 (14) | 13 (9) | <0.001 |
| Dysphagia | 17 (27) | 37 (23) | 5 (55) | 13 (37) | 62 (44) | <0.001 |
| Alopecia | 10 (15) | 9 (5) | 3 (33) | 3 (8) | 22 (15) | 0.016 |
| Interstitial Lung Disease | 36 (57) | 19 (11) | 1 (11) | 3 (8) | 15 (10) | <0.001 |
| Cardiac Involvement | 1 (1) | 4 (2) | 1 (11) | 0 (0) | 3 (2) | 0.418 |
| ^1^Chi-squared test | | | | | | |

**Supplementary Table S10** Malignancies by disease cohort

| **Location of malignancy n (%)** | **DM (n=405)** | **ASyS (n=649)** | **ASyS-DMskin**  **(n=203)** | **ASyS-without-DMskin**  **(n=446)** | **DM vs ASyS**  **Adjusted p-value** | **DM vs ASyS-DMskin**  **Adjusted p-value** | **ASyS-DMskin vs ASyS-without-DMskin**  **Adjusted**  **p-value** |
| --- | --- | --- | --- | --- | --- | --- | --- |
| Bladder | 5 (1) | 2 (0.3) | 0 (0) | 2 (0.4) | 0.411 | 0.689 | 1 |
| Bowel | 12 (3) | 2 (0.3) | 1 (0.5) | 1 (0.2) | 0.013 | 0.396 | 1 |
| Breast | 16 (4) | 7 (1) | 3 (2) | 4 (1) | 0.017 | 0.52 | 1 |
| Hepatic | 1 (0.2) | 0 (0) | 0 (0) | 0 (0) | 1 | 1 | 1 |
| Kidney | 0 (0) | 0 (0) | 0 (0) | 0 (0) | 1 | 1 | 1 |
| Lung | 10 (3) | 3 (0.5) | 0 (0) | 3 (0.7) | 0.032 | 0.357 | 1 |
| Lymphoma | 2 (0.5) | 3 (0.5) | 1 (0.5) | 2 (0.4) | 1 | 1 | 1 |
| Melanoma | 1 (0.2) | 0 (0) | 0 (0) | 0 (0) | 1. | 1 | 1 |
| Ovarian | 15 (4) | 0 (0) | 0 (0) | 0 (0) | 0.007 | 0.161 | 1 |
| Pancreas | 1 (0.2) | 0 (0) | 0 (0) | 0 (0) | 1 | 1 | 1 |
| Prostate | 4 (1) | 2 (0.3) | 0 (0) | 2 (0.4) | 1 | 0.695 | 1 |
| Uterine | 5 (1) | 4 (0.6) | 2 (1) | 2 (0.4) | 1 | 1 | 1 |
| Other | 6 (2) | 11 (1.7) | 6 (3) | 5 (1) | 1 | 0.771 | 1 |

**Supplementary Table S11** Malignancies by antibody cohort

|  | **EJ**  **(n=16)** | **Jo1**  **(n=541)** | **MDA5**  **(n=63)** | **MI2**  **(n=160)** | **NXP2**  **(n=9)** | **OJ**  **(n=10)** | **PL12**  **(n=41)** | **PL7**  **(n=35)** | **SAE**  **(n=35)** | **TIF1γ**  **(n=138)** | **Zo (n=6)** | **Adjusted p-value^1^** |
| --- | --- | --- | --- | --- | --- | --- | --- | --- | --- | --- | --- | --- |
| Bladder | 0 (0) | 0 (0) | 0 (0) | 0 (0) | 0 (0) | 0 (0) | 2 (4.9) | 0 (0) | 2 (5.7) | 3 (2.2) | 0 (0) | <0.001 |
| Bowel | 0 (0) | 1 (0.2) | 0 (0) | 7 (4.4) | 0 (0) | 0 (0) | 1 (2.4) | 0 (0) | 1 (2.9) | 4 (2.9) | 0 (0) | 0.014 |
| Breast | 0 (0) | 5 (0.9) | 1 (1.6) | 4 (2.5) | 0 (0) | 0 (0) | 2 (4.9) | 0 (0) | 1 (2.9) | 10 (7.2) | 0 (0) | 0.008 |
| Hepatic | 0 (0) | 0 (0) | 0 (0) | 0 (0) | 0 (0) | 0 (0) | 0 (0) | 0 (0) | 0 (0) | 1 (0.7) | 0 (0) | 0.759 |
| Kidney | 0 (0) | 0 (0) | 0 (0) | 0 (0) | 0 (0) | 0 (0) | 0 (0) | 0 (0) | 0 (0) | 0 (0) | 0 (0) | 1 |
| Lung | 0 (0) | 3 (0.6) | 1 (1.6) | 0 (0) | 0 (0) | 0 (0) | 0 (0) | 0 (0) | 0 (0) | 9 (6.5) | 0 (0) | <0.001 |
| Lymphoma | 0 (0) | 3 (0.6) | 0 (0) | 0 (0) | 0 (0) | 0 (0) | 0 (0) | 0 (0) | 0 (0) | 2 (1.4) | 0 (0) | 0.914 |
| Melanoma | 0 (0) | 0 (0) | 0 (0) | 0 (0) | 0 (0) | 0 (0) | 0 (0) | 0 (0) | 0 (0) | 1 (0.7) | 0 (0) | 0.759 |
| Ovarian | 0 (0) | 0 (0) | 0 (0) | 1 (0.6) | 0 (0) | 0 (0) | 0 (0) | 0 (0) | 1 (2.9) | 13 (9.4) | 0 (0) | <0.001 |
| Pancreas | 0 (0) | 0 (0) | 0 (0) | 1 (0.6) | 0 (0) | 0 (0) | 0 (0) | 0 (0) | 0 (0) | 0 (0) | 0 (0) | 0.848 |
| Prostate | 0 (0) | 1 (0.2) | 0 (0) | 0 (0) | 0 (0) | 0 (0) | 0 (0) | 1 (2.9) | 2 (5.7) | 2 (1.4) | 0 (0) | 0.006 |
| Uterine | 0 (0) | 4 (0.7) | 0 (0) | 0 (0) | 0 (0) | 0 (0) | 0 (0) | 0 (0) | 0 (0) | 5 (3.6) | 0 (0) | 0.105 |
| Other | 0 (0) | 11 (2.0) | 1 (1.6) | 3 (1.9) | 0 (0) | 0 (0) | 0 (0) | 0 (0) | 1 (2.9) | 1 (0.7) | 0 (0) | 0.963 |
| **CAM n (%)** | 0 (0) | 17 (3.1) | 2 (3.2) | 12 (7.5) | 0 (0) | 0 (0) | 3 (7.3) | 1 (2.9) | 7 (20.0) | 46 (33.3) | 0 (0) | <0.001 |
| ^1^Chi-squared test | | | | | | | | | | | | |
